# Uptake of the Necrotic Serpin in *Drosophila melanogaster* via the Lipophorin Receptor-1

**DOI:** 10.1371/journal.pgen.1000532

**Published:** 2009-06-26

**Authors:** Sandra Fausia Soukup, Joaquim Culi, David Gubb

**Affiliations:** 1 Functional Genomics Unit, CIC bioGUNE, Derio, Spain; 2 Centro Andaluz de Biología del Desarrollo (CSIC-UPO), Universidad Pablo de Olavide, Sevilla, Spain; University of California San Francisco, United States of America

## Abstract

The humoral response to fungal and Gram-positive infections is regulated by the serpin-family inhibitor, Necrotic. Following immune-challenge, a proteolytic cascade is activated which signals through the Toll receptor. Toll activation results in a range of antibiotic peptides being synthesised in the fat-body and exported to the haemolymph. As with mammalian serpins, Necrotic turnover in *Drosophila* is rapid. This serpin is synthesised in the fat-body, but its site of degradation has been unclear. By “freezing” endocytosis with a temperature sensitive Dynamin mutation, we demonstrate that Necrotic is removed from the haemolymph in two groups of giant cells: the garland and pericardial athrocytes. Necrotic uptake responds rapidly to infection, being visibly increased after 30 mins and peaking at 6–8 hours. Co-localisation of anti-Nec with anti-AP50, Rab5, and Rab7 antibodies establishes that the serpin is processed through multi-vesicular bodies and delivered to the lysosome, where it co-localises with the ubiquitin-binding protein, HRS. Nec does not co-localise with Rab11, indicating that the serpin is not re-exported from athrocytes. Instead, mutations which block late endosome/lysosome fusion (dor, hk, and car) cause accumulation of Necrotic-positive endosomes, even in the absence of infection. Knockdown of the 6 *Drosophila* orthologues of the mammalian LDL receptor family with dsRNA identifies LpR1 as an enhancer of the immune response. Uptake of Necrotic from the haemolymph is blocked by a chromosomal deletion of LpR1. In conclusion, we identify the cells and the receptor molecule responsible for the uptake and degradation of the Necrotic serpin in *Drosophila melanogaster*. The scavenging of serpin/proteinase complexes may be a critical step in the regulation of proteolytic cascades.

## Author Summary

Serpin inhibitors control a wide range of rapid physiological responses that are activated by proteolytic cascades, such as blood coagulation, inflammation, the complement pathway, and angiogenesis. They interact with their target proteinases by a “suicide inhibition” mechanism, which generates an inert, denatured, serpin/proteinase complex. In mammals, humoral serpins are secreted from the liver into the blood plasma. The denatured complex is later endocytosed back into the liver and degraded. In *Drosophila*, the Necrotic serpin is secreted from the fat-body into the haemolymph, where it controls the humoral immune response. We show here, however, that Necrotic is not endocytosed in the fat-body, but in the garland and pericardial athrocytes. These cells clear serpins from the haemolymph extremely rapidly. The Necrotic-binding receptor for this process is LpR1, a member of the LDLR family. The endocytosed serpin is targeted for lysosomal degradation, with none being recycled to the haemolymph. More importantly, we show that mutations in LpR1 cause a profound effect on the immune response. Thus, our results indicate that the scavenging of serpin/proteinase complexes might be a critical step in the regulation of proteolytic cascades.

## Introduction

The immune response to pathogen challenge in humans consists of an immediate “innate” response (mediated via cellular responses and the action of antimicrobial peptides), followed by a delayed “acquired” response (mediated by antibodies). In insects, the antibody response is absent, but these organisms synthesise antibiotic peptides, activate macrophage-like cells and mount a melanization response [1]. The *necroti*c (*nec*) gene encodes a proteinase inhibitor of the serpin family (**ser**ine **p**roteinase **in**hibitor). Nec controls a proteolytic cascade which activates the innate immune response to fungal and Gram^+^ bacterial infections [2]. The Nec serpin carries an N-terminal extension which modifies its substrate specificity and is cleaved following immune-challenge [3]. In *nec* null mutants, the Toll-mediated immune response is constitutively activated, even in the absence of infection, implying that Nec continually restrains this immune response. The serpins have been extensively studied in mammals, where they regulate many extracellular proteolytic cascades. The coagulation, inflammatory and complement pathways are controlled by α_1_-Antithrombin, α_1_-Antitrypsin and C1-Inhibitor, respectively [4]–[6]; while Plasminogen Activator Inhibitor-1 modulates angiogenesis, affecting both wound-healing and tumour growth [7]. Disorders in serpin metabolism underlie a major group of human genetic diseases, the serpinopathies, which are associated with failure to clear inert serpin polymers [5],[8],[9] and homologous mutations in Necrotic similarly form inactive polymers [10],[11].

Serpins interact with their target proteinase via a “suicide-inhibition” mechanism, which destroys both serpin and proteinase and generates a covalently-linked complex [12]. Inert serpin/proteinase complexes are removed from circulation by endocytosis in the liver [13], via receptors of the low-density lipoprotein (LDLR) family [14],[15]. The LDLR family consists of a diverse group of cell surface receptors [16] that is evolutionarily conserved [17]. LDLR/ligand binding is pH-dependent, so that the complex dissociates in the low pH environment of the endosomal compartment, allowing LDLR to be recycled to the cell surface [18]–[20].

During endocytosis, the internalization of receptor-bound proteins requires Dynamin function for the pinching-off of clathrin-coated vesicles [21]. Endocytosed proteins are transported to various intracellular compartments, with the Rab family of Ras-related GTPases being critical for coordinating vesicle formation, transport and fusion with the target membrane [22]. In particular, maturation of the early endosome coincides with the replacement of Rab5 by Rab7 and the accumulation of lumenal vesicles to form multivesicular bodies (MVB) [23]. MVB correspond to a class of late endosome, which requires Hook and Fab1 for maturation [24]. Following LDLR/ligand dissociation, transport of the free LDLR back to the plasma membrane is mediated via Rab11-positive, recycling endosomes. The contents of MVBs are delivered by direct fusion, either to lysosomes, or to the plasma membrane [25]. A key component in protein sorting from late endosomes to lysosomes is ubiquitination. In this process, HRS (hepatocyte growth factor-regulated kinase substrate) binds ubiquitin and interacts with ubiquitinated cargos in the early endosome [26], while *Fab1* encodes a phosphatidylinositol(3)-phosphate 5-kinase which acts downstream of HRS [27],[28]. In *Drosophila*, MVB fusion with the lysosome requires the function of the *deep orange* (*dor*) and *carnation* (*car*) subunits of the HOPS (homeotypic vacuole fusion and protein sorting) trafficking complex [29].

The garland cells represent an attractive model system to study endocytotic processes, since they are giant cells that are highly active in clathrin-mediated endocytosis. They form two rows of loosely-connected cells surrounding the oesophagus at the junction with the proventriculus, but little is known about their biological function [30]–[33]. Garland cells are bi-nucleated with a highly vacuolated structure featuring deep invaginations of the plasma membrane [34]. The pericardial cells, on the other hand, are arranged in two rows of approximately 20 cells, on either side of the heart. Both cell types have a similar morphology and express the WD40 domain protein, Rudhira [34],[35] and the *dot-Gal4* enhancer trap [36], while their positioning around the proventriculus and dorsal vessel ensures good contact with flowing haemolymph. In contrast to the garland cells, pericardial cells are mono-nucleate; although both cell types are bi-nucleate in *Hyalophora cecropia* and *Calliphora erythrocephala* [37],[38]. These two cell types are homologous to athrocytes in other insects [39] and share a nephrin/podocin based filtration mechanism with the vertebrate kidney glomerulus [40]. Instead of being passed to a nephric tubule, however, the ultrafiltrate is endocytosed from lacunae in the garland and pericardial cells [34],[41],[42]. There is no connection between these athrocytes and the Malpighian tubules, which regulate the concentration of plasma ions and metabolites in Insects. In this respect, the garland and pericardial cell clusters resemble more the vertebrate reticulo-endothelial cells [41],[43] than the kidney glomerulus. The reticulo-endothelial system consists of groups of phagocytic cells that take up denatured proteins, bacterial and viral components [44]–[46].

In this study, we investigate the role of LDL-family receptors in the Toll-mediated immune response of *Drosophila* by dsRNA-knockout. We have tested the role of the Lipophorin Receptors, LpR1 and LpR2, in Nec processing. We show that Nec is taken up in the garland and pericardial cells by LpR1, probably as a serpin/proteinase complex. Antibody staining against different proteins involved in clathrin-mediated endocytosis and intracellular trafficking establishes that Nec is sorted and degraded in the garland and pericardial athrocytes, but not recycled to the haemolymph. In general, the paucity of Rab11-positive vesicles indicates that these cells do not recycle endocytotic components, but are the site of protein and peptide degradation.

## Results

### Necrotic expression pattern and activation by infection

Northern-blot analysis showed that *nec* is expressed at low levels in larvae and moderate/high levels in pupae and adults, in unchallenged flies [10]. Here we use quantitative RT-PCR to quantify *nec* transcript levels with and without infection. We found no *nec* expression in embryos, moderate levels in larvae and adults, and higher levels in pupal stages. Six hours after infection with *M. luteus*, *nec* transcript was up-regulated 10 fold in larvae and 7 fold in adults, to reach similar levels (Figure S1). Nec expression was induced 4–6 fold after septic injury with *Micrococcus luteus* and 3.4 fold after fungal infection with *Beauvaria bassiana* in adult flies [47]. Given these results, we analysed Nec uptake after infection in both larvae and adults.

*In situ* hybridization showed strong *nec* expression in the fat-body of infected wild-type larvae, which is lost in *nec* transcript null larvae (Figure S2). In addition, *nec* transcript was detected in mid- and hind-gut. We did not detect *nec* transcript in Malpighian tubules, imaginal discs, brain tissue, pericardial or garland cells (Figure S2) of wild-type larvae. These results are in broad agreement with expression array data from the FlyAtlas (http://flyatlas.org/atlas). The anti-Nec antibody detects protein staining in the fat-body of wild-type larvae, which is lost in protein null larvae (data not shown).

### Nec is taken up from the haemolymph by the garland cells

We were initially unable to detect Nec protein in any larval or adult tissues, apart from the fat-body, using a rabbit polyclonal antibody that works on Western blots. Similar experiments using an antibody to Serpin27A also failed to detect antibody staining outside the fat-body. These preliminary findings are consistent with the hypothesis that serpin turnover in the haemolymph is rapid and that tissue-fixation might be slower than serpin degradation. To test this hypothesis, we blocked the formation of clathrin-coated vesicles, using the temperature-sensitive Dynamin mutation, *shi^ts1^* [30]. Third instar *shi^ts1^* larvae were grown, infected and dissected at the permissive temperature of *shi^ts1^* and shifted to the restrictive temperature during fixation. This procedure combines rapid fixation with a block in endocytosis.

**Figure 1:**
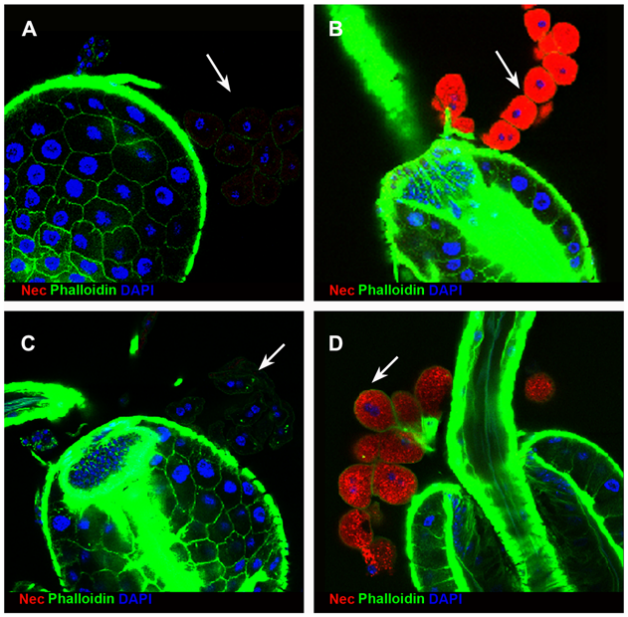
Nec is detected in *shi^ts1^* garland cells. (A) Wild-type garland cells (white arrow) 6 h post infection. (B) *shi^ts^* mutant 6 h post infection showing strong Nec localisation (red) to garland cells (white arrow). (C) Wild-type garland cells (white arrow) without infection. (D) *shi^ts^* mutant garland cells (white arrow) without infection show faint, basal Nec levels. The ring of garland cells is loosely inter-connected with fine Actin fibres, which frequently break during preparation. All preparations were fixed at 37°C. Nec (red), Actin (green), and DAPI (DNA) (blue).

Under these conditions, Nec antibody staining was absent, or barely detectable, in the garland cells of infected wild-type larvae, but strong staining was seen in the garland cells of infected *shi^ts1^* larvae (Figure 1). The basal level of Nec expression was detected, at reduced intensity, in uninfected *shi^ts1^* larvae (Figure 1D). Similar results were seen in pericardial cells and in adult stages. The specificity of the Nec antibody was confirmed by the loss of protein staining in a *shi^ts^*; *nec* null background (Figure S2D). Given that *in situ* hybridization failed to detect *nec* transcript (Figure S2C), the Nec protein in garland cells is presumably taken-up from the haemolymph. RNAi knockdown of *nec* transcript in the garland cells (of *shi^ts^*; *UAS-nec^dsRNAi^*; *dot-Gal4 UAS-GFP* larvae) has no effect on Nec antibody staining, which remained strong (Figure S3). Note that garland cells bud-off endocytotic vesicles from labyrinthine channels, within a cortical region of several microns depth, rather than from the outer cell surface [30],[31]. As a result, blocking endocytosis leaves the walls of the labyrinthine channels coated with bound Nec protein.

To confirm that the Nec visualised in garland cells derives from protein synthesis in the fat-body we knocked down *nec* transcription in the fat-body, in *shi^ts^*; *UAS-nec^dsRNAi^/c564-Gal4* larvae. Garland cells from these larvae show no detectable Nec protein (Figure S4).

Taken together, these results indicate that garland and pericardial cells take-up Nec from the haemolymph and confirm that this protein is synthesised in the fat-body.

### Time-course of Nec uptake in response to infection

The intensity of Nec-antibody staining in garland cells showed a rapid increase after infection with *M. luteus*, which is visible by 30 mins post-infection, peaks between 6 and 8 h and returns to basal levels within 24 h (Figure S5).

### The Nec endocytotic pathway

#### Nec is endocytosed in clathrin-coated vesicles

**Figure 2:**
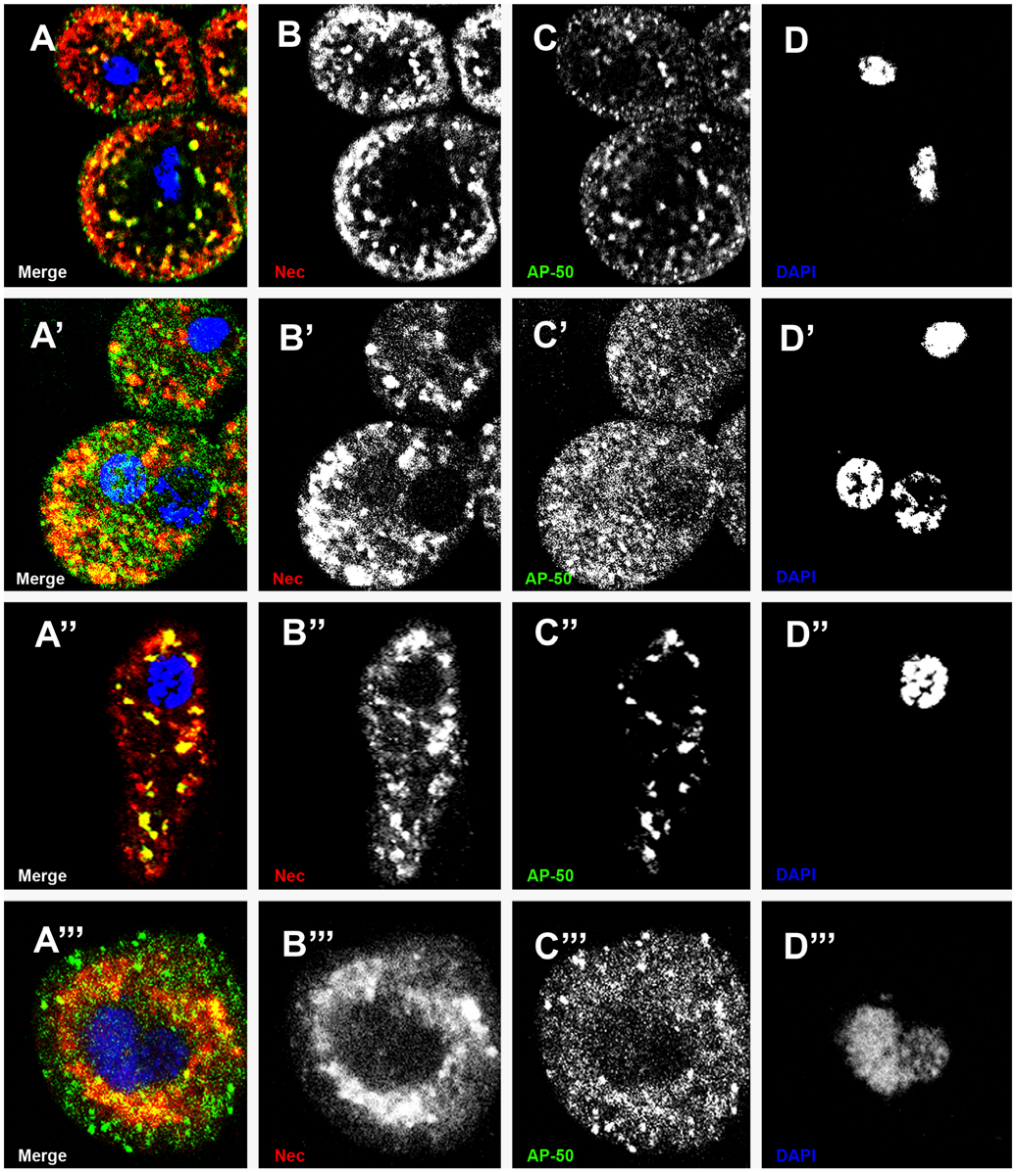
Partial co-localisation of Nec in clathrin-coated vesicles in garland and pericardial cells. (A) larval garland cells, (A′) adult garland cells, (A″) larval pericardial cells and (A′″) adult pericardial cells merge of single channels showing co-localization of Nec and AP50 (yellow), Nec^+^ AP50^−^ vesicles (red) and Nec^−^ AP50^+^ vesicles (green). (B, B″) larval stage and (B′, B′″) adult stage Nec channel. (C, C″) larval stage and (C′, C′″) adult stage AP50 channel. (D, D″) larval stage and (D′,D′″) adult stage DAPI channel. Immunostainings were 6 hours post infection in a *shi^ts1^* background. Nec (red), AP-50 (green), and DAPI (blue).

Clathrin-coated vesicles co-localised with Nec in garland and pericardial cells, in both larval and adult stages (Figure 2). Many vesicles stained AP50-positive (clathrin-coated) and Nec-positive; but some AP50^−^ Nec^+^ and AP50^+^ Nec^−^ vesicles were also seen. These results indicate that Nec is internalized in clathrin-coated vesicles and presumably re-sorted to other, clathrin-negative, compartments.

#### Nec is sorted through Rab-positive endosomes for degradation

**Figure 3:**
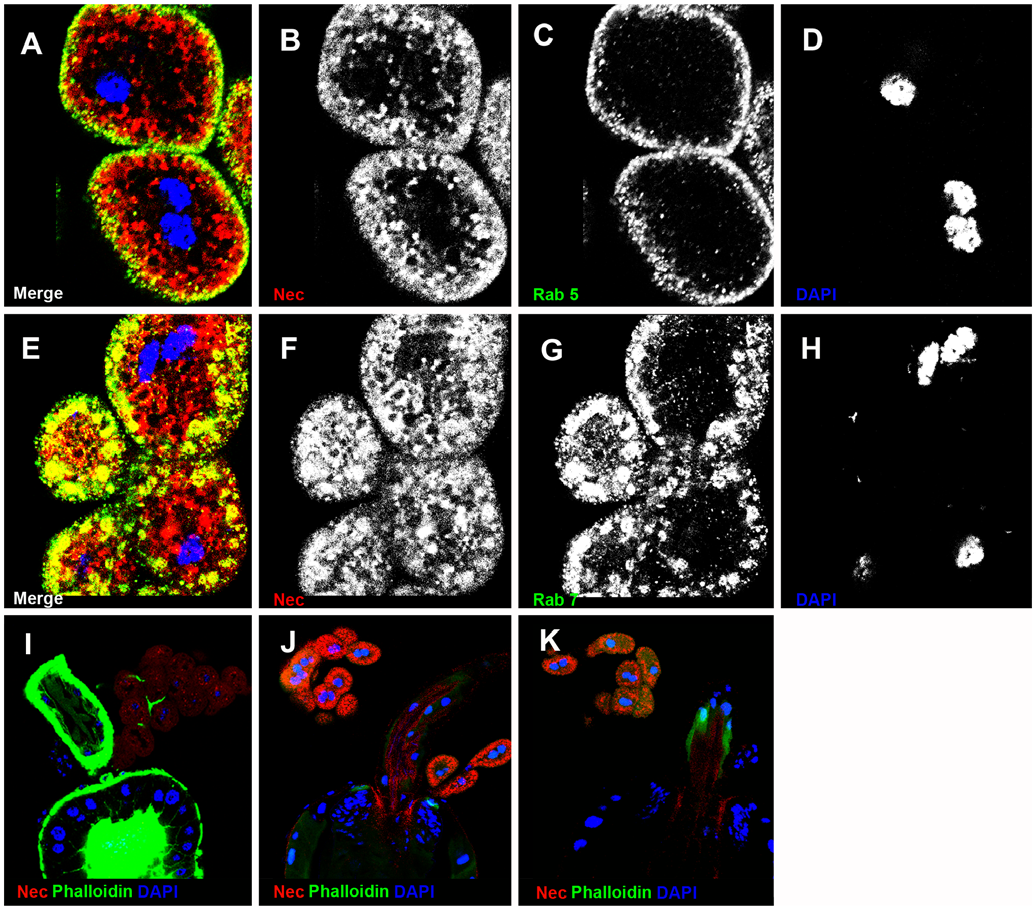
Nec is sorted into Rab5- and Rab7-positive endosomes in garland cells. (A) Merge of B–D shows co-localization of Nec and Rab5 in yellow. (B) Nec channel. (C) Rab5 channel. (D) DNA channel. (E) Merge of B–D shows co-localization of Nec and Rab7 in yellow (F) Nec channel. (G) Rab7 channel. (H) DNA channel. (I) *shi^ts1^* flies without infection. (J) *UAS-Rab5^S43N^* mutant flies without infection. (K) *UAS-Rab7^Q67L^* mutant flies without infection. Nec (red), Rab5 (green in A and B) Rab7 (green in E and G), and DAPI (blue).

Rab5 antibody staining partially co-localizes with Nec-positive vesicles in early endosomes (Figure 3A). Driving mutant *UAS-Rab5^S43N^* expression in garland cells, under the control of *dot-Gal4*, resulted in strong accumulation of Nec-positive vesicles (Figure 3J) even in the absence of infection. Similarly, Nec partially co-localized with Rab7-positive late endosomes (Figure 3E) and expressing *UAS-Rab7^Q67^* mutant protein lead to accumulation of Nec-positive vesicles in the absence of infection (Figure 3K). The *UAS*-*Rab5^S43N^* and *UAS*-*Rab7^Q67L^* transgenic constructs caused a similar block in Nec processing. Taken together, these results suggest that Rab5 and Rab7 are critical for Nec sorting and degradation in garland cells.

#### Nec requires sorting through multi-vesicular bodies for degradation

**Figure 4:**
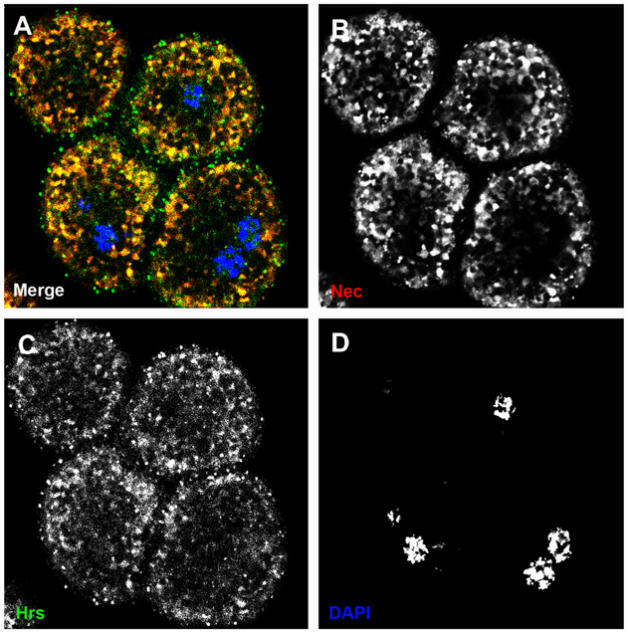
HRS and Nec are sorted in the same early endosomes. (A) Merge shows co-localization of HRS and Nec in yellow. (B) Nec channel. (C) HRS channel. (D) DNA channel. Nec (red), HRS (green), and DAPI (blue).

From the early endosome, the endocytotic cargo is sorted into multivesicular bodies and can be delivered to the lysosome for degradation. HRS antibody staining was used to identify ubiquitinated endosomes, in the sorting pathway from the early endosome [26] to multivesicular bodies. As shown in Figure 4A, Nec staining co-localizes with HRS staining in early endosomes.

**Figure 5:**
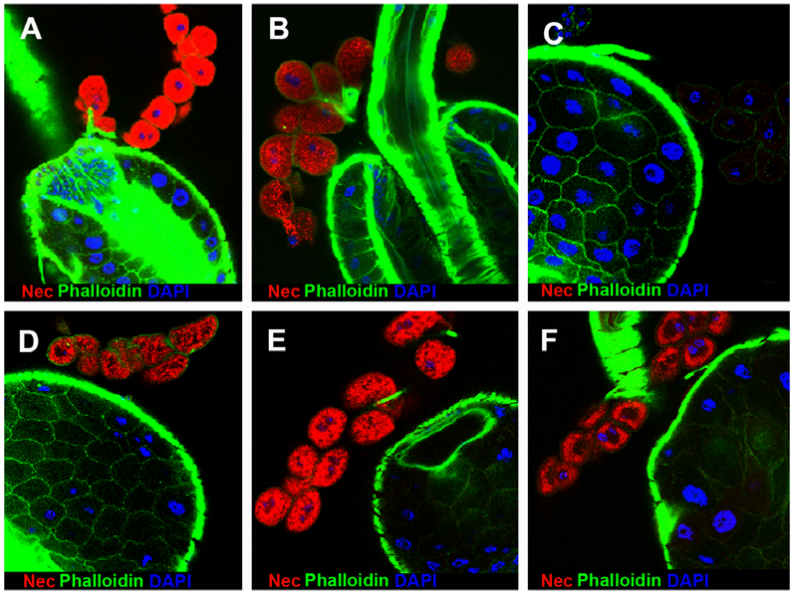
Mutations in the HOPS complex block Nec-sorting through the multivesicular body and lysosomal delivery. (A) *shi^ts1^*6h post infection. (B) *shi^ts1^* without infection. (C) Wild-type 6 h post infection. (D) *dor^1^/dor^8^* mutant without infection. (E) *car^1^* mutant without infection. (F) *hk^1^* mutant without infection. Nec (red), Actin (green), and DAPI (blue).

To investigate lysosomal delivery, we used the *dor*, *hook* and *car* mutants of the HOPS complex to block late endosome/lysosome fusion [29],[48],[49]. In all three mutants, Nec-positive endosomes/MVB accumulate even in the absence of infection (Figure 5). We conclude therefore that Nec sorting through MVBs via the HOPS complex is required for lysosomal delivery.

#### Nec co-localises with Fab1

**Figure 6:**
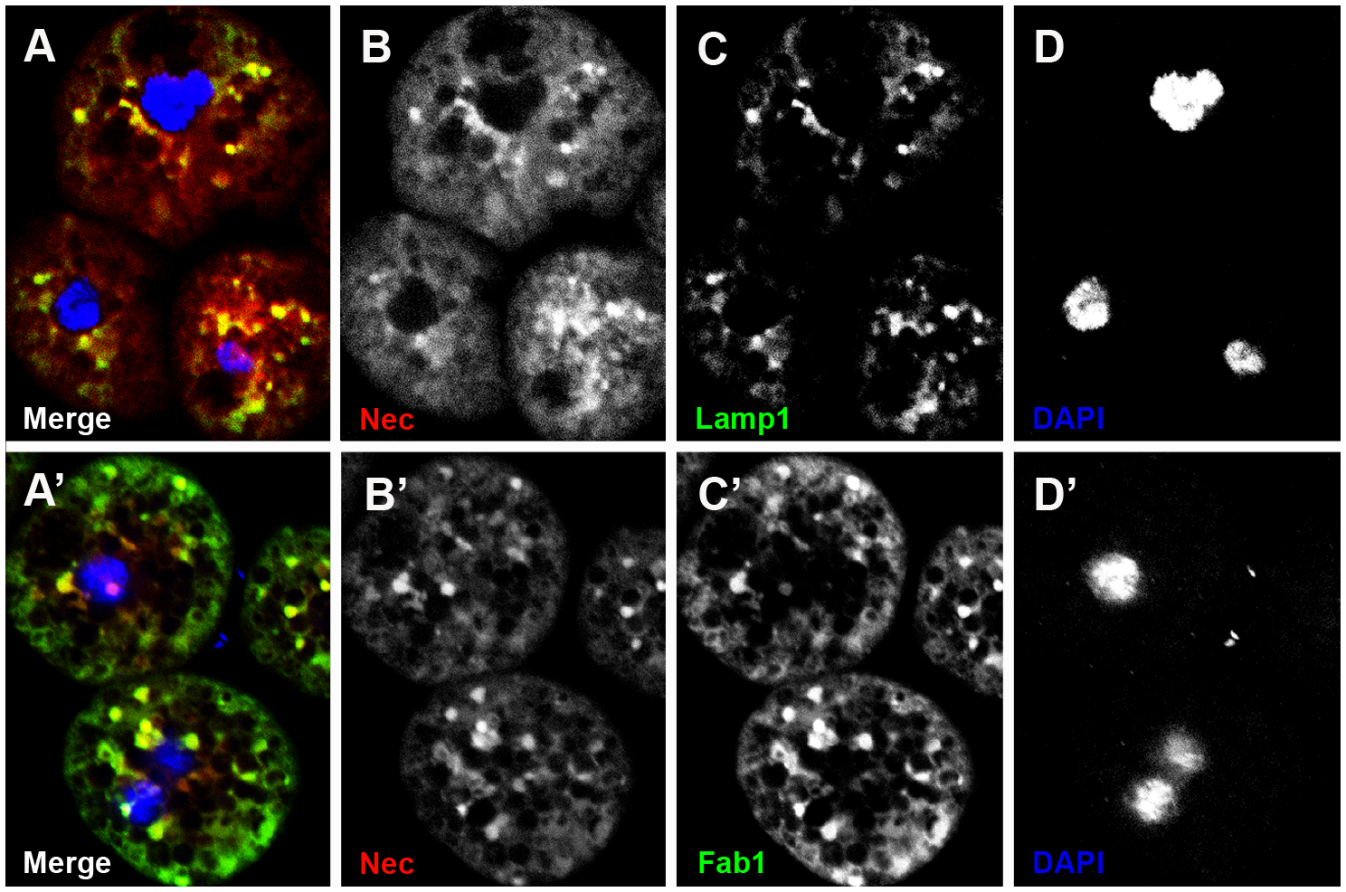
Nec co-localizes with Fab1 and Lamp1 in garland cells. (A–D) Fab1 co-localizes with Nec in garland cells. (A) Merge shows co-localization of Lamp1 and Nec in yellow. (B) Nec channel. (C) Lamp1 channel. (D) DAPI channel. (A′–D′) Fab1 co-localizes with Nec in garland cells. (A′) Merge shows co-localization of Fab1 and Nec in yellow. (B′) Nec channel. (C′) Fab1 channel. (D′) DNA channel. Nec (red), Lamp1 (green in A and C), Fab1 (green in A′ and C′), and DAPI (blue).

Fab1 acts downstream of HRS [27],[28] and is required for vacuolar membrane trafficking and delivery to the lysosome. Antibody staining shows partial co-localization of Fab1 and Nec, indicating that Nec is destined for lysosomal degradation by Fab1 (Figure 6).

#### Internalised Nec is targeted for lysosomal degradation and is not re-exported to the haemolymph

An antibody to a lysosomal membrane protein, Lamp1, was used to confirm that Nec is trafficked to the lysosomal compartment. Co-localisation of Nec and Lamp1 antibody staining confirms that Nec is sorted from MVB to lysosomes, where it is degraded (Figure 6).

**Figure 7:**
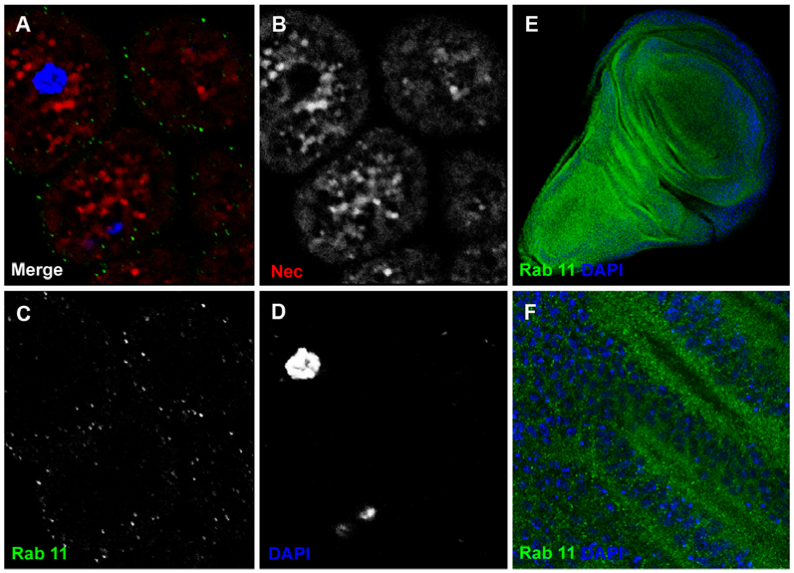
Rab11-positive vesicles are much less prominent in garland cells than in the wing disc. (A) Merge shows distinct populations of Rab11-positive vesicles (green) and Nec-positive vesicles (red). (B) Nec channel. (C) Rab11 channel. (D) DAPI channel. Nec (red), Rab11 (green) and DAPI (blue) (E) larval wing discs with Rab11 and DAPI staining. (F) magnification of E.

An alternative trafficking pathway for proteins within MVBs is that they can be re-exported to the haemolymph via Rab11-positive recycling vesicles [50]. We found that garland cells contain few Rab11-positive vesicles in general, and that the Nec protein staining did not co-localize with those Rab11-positive vesicles that were present (Figure 7). Therefore, the Nec protein taken-up by the garland cells is targeted exclusively for lysosomal degradation.

#### Identification of the Nec trafficking receptor *dsRNA* knockdown assays

The 6 *Drosophila* orthologues of the mammalian LDLR family were tested for an immune response function by dsRNA knockdown. Adult flies were injected with LDLR dsRNAs and 4 days later infected with *M. luteus* [51]. Toll pathway activity was assayed by quantifying transcript levels of the antibiotic peptide Drosomycin (Drs), (Figure S6). Knockdown efficiencies for the *D*-LDLR orthologues were: *LpR2* (92%), *LpR1* (86%), *CG33087* (87%), *CG12139* (91%), *arrow* (56%) and *CG8909* (88%), as estimated by qRT-PCR. Under these conditions, silencing of *CG33087*, *CG12139*, *arrow* and *CG8909* does not affect *Drs* transcript levels, with a barely significant reduction with silencing of *LpR2*. Strikingly, *LpR1*-silencing doubles *Drs* transcript levels. This result implies that partial knockdown of *LpR1* decreases Nec activity in the haemolymph. To confirm this result we measured *nec* and *Drs* transcript levels in *Df(3R)lpr1*, *Df(3R)lpr2* and *Df(3R)lpr1/2* in infected flies (Figure S7). Deficiency of *LpR1* reduces *nec* (58%) and increases *Drs* transcript levels (206%), while deficiency of *LpR2* has a weaker effect in the opposite direction. In the absence of immune challenge, *Drs* transcript levels are (1030%) higher in *Df(3R)lpr1* than in wild-type flies (Figure S7).

### Chromosomal deletion of *LpR1* blocks Nec uptake into garland cells

**Figure 8:**
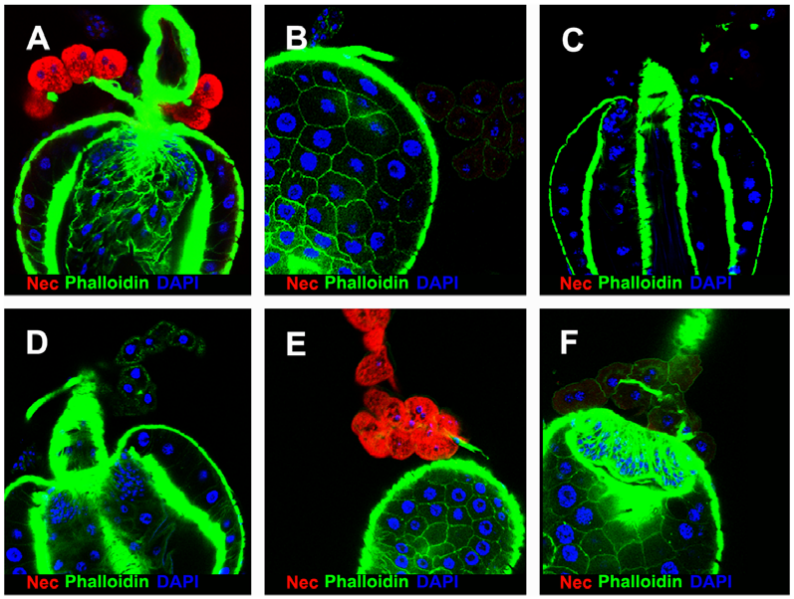
*LpR1* is the Receptor for Nec uptake in immune-challenged garland cells. (A–F) larval garland cells 6 h post infection (A) *shi^ts1^* mutant. (B) Wild-type. (C) *shi^ts1^*; *Df(3R)lpr1*. (D) *shi^ts1^*; *pBac(LpR1)*. (E) *shi^ts1^*; *Df(3R)lpr2* (F) *shi^ts1^*; *Df(3R)lpr1/2*. All pictures captured with same setting and laser intensity. Nec (red), Actin (green), and DAPI (blue).

The *LpR1* and *LpR2* genes are adjacent transcripts in Drosophila. Flies carrying either a chromosomal deletion for *LpR1*, (*Df(3R)lpr1*), the *pBac(LpR1)* mutation, or a deletion of both *LpR* transcripts (*Df(3R)lpr1/2*), fail to take up Nec into garland cells. Conversely, deletion of the *LpR2* transcript only (in *Df(3R)lpr2* flies) leaves Nec uptake unaffected (Figure 8).

### LpR1, LpR2, and Nec co-localize in garland and pericardial cells

**Figure 9:**
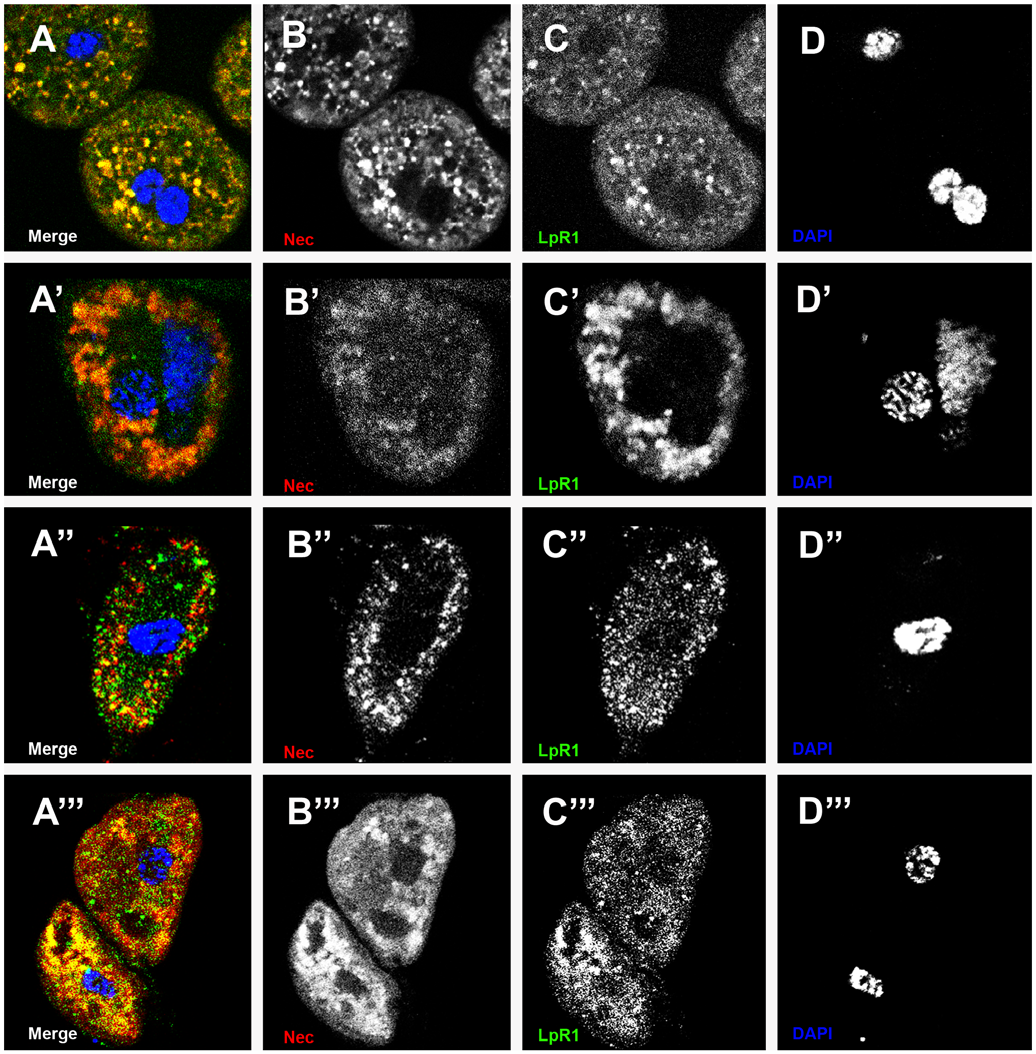
LpR1 and Nec co-localize in garland cells and pericardial cells. (A–D) larval, (A′–D′) adult garland cells, (A″–D″) larval and (A′″–D′″) adult pericardial cells, 6 h post infection. (A, A′, A″, and A″′) Merge shows LpR1 and Nec co-localizing (yellow) in endosomes. (B, B′, B″, and B′″) Nec channel. (C, C′, C″, and C′″) LpR1 channel. (D, D′, D″, and D′″) DNA channel. Nec (red), LpR1 (green), and DAPI (blue).

Antibody staining of LpR1 and Nec in larval and adult garland and pericardial cells showed co-localisation (Figure 9). The equivalent experiments with LpR2 antibody showed that this receptor also co-localises with Nec (Figure S8). Taken together with the blocking of Nec uptake in *Df(3R)lpr1* flies, these results confirm that LpR1 is the Nec trafficking receptor and that LpR2 is present, but does not traffic Nec, under these conditions.

### The LpR1 receptor is required for efficient uptake of a Nec-proteinase complex

**Figure 10:**
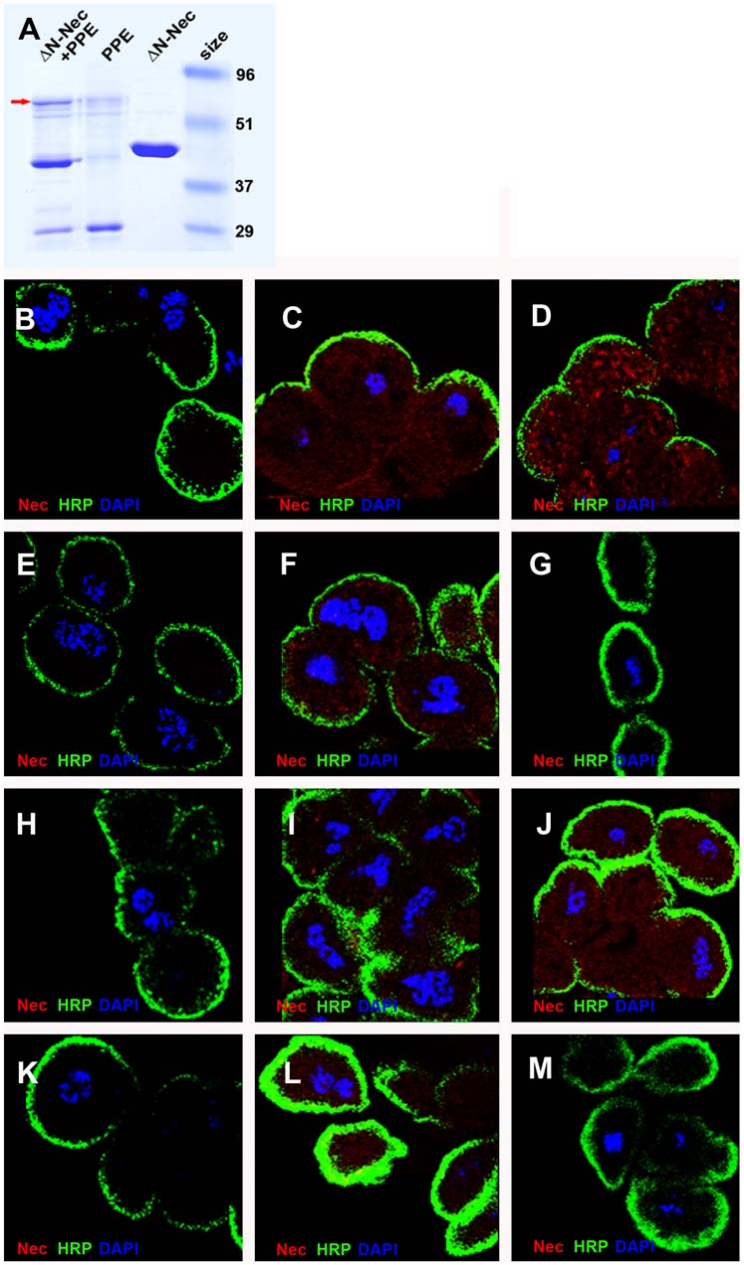
Endocytotic assay of Nec monomer and Nec-complex in garland cells. (A) Formation of Nec-PPE complex. Coomassie blue stained PAGE gel showing PPE digest of Nec^ΔN^. The digestion reduces the intensity of Nec^ΔN^ band to 20% and 8% of Nec is found in the complex band (arrow). The uptake of Nec^ΔN^-PPE complex uptake is blocked by the *LpR1* and *LpR1-2* deletions, but not the *LpR2* deletion. Native Nec uptake is decreased by the *LpR2* and *LpR1-2* deletions. (B) *shi^ts1^*+PBS control; (C) *shi^ts1^*+Nec^ΔN^; (D) *shi^ts1^*+Nec^ΔN^/PPE complex. (E) *shi^ts1^*; *Df(3R)lpr1*+PBS control; (F) *shi^ts1^*; *Df(3R)lpr1*+Nec^ΔN^; (G) *shi^ts1^*; *Df(3R)lpr1*+Nec^ΔN^/PPE complex. (H) *shi^ts1^*; *Df(3R)lpr2*+PBS control; (I) *shi^ts1^*; *Df(3R)lpr2*+Nec^ΔN^; (J) *shi^ts1^*; *Df(3R)lpr2*+Nec^ΔN^/PPE complex. (K) *shi^ts1^*; *Df(3R)lpr1/2*+PBS control; (L) *shi^ts1^*; *Df(3R)lpr1/2*+Nec^ΔN^; (M) *shi^ts1^*; *Df(3R)lpr1/2*+Nec^ΔN^/PPE complex. All samples were prepared together and pictures captured with same confocal settings and laser intensity. Nec (red), HRP (cell membrane) (green), DAPI (blue).

Digestion of the Nec^ΔN^ core serpin (lacking the N-terminal peptide) with porcine pancreatic elastase (PPE) produces a covalently linked Nec^ΔN^-PPE complex with reduced levels of native Nec^ΔN^ (Figure 10A). This native serpin+serpin/protease complex mixture was taken up more readily by garland cells than the pure Nec^ΔN^ sample, provided that the *LpR1* transcript was present. Nec^ΔN^-PPE complex uptake was blocked by the *Df(3R)lpr1* and *Df(3R)lpr1/2* chromosomal deletions. In addition, uptake of the undigested, native Nec^ΔN^, serpin was decreased by the *Df(3R)lpr2* and *Df(3R)lpr1/2* deletions (Figure 10G and 10J). These results are consistent with LpR1 being the receptor for the inert Nec-proteinase complex and *LpR2* being the main receptor for native Nec, although other receptors may well be responsible for some of the native Nec binding.

## Discussion

The Necrotic serpin controls activation of the Toll-mediated immune-response in *Drosophila*, which represents the best-studied example of serpin-regulated proteolytic cascade in insects [2],[3],[52]. The *Drosophila melanogaster* genome encodes 15 putative inhibitory serpin transcripts that carry secretion-signal peptides [53]. In addition to *nec*, the *Spn27A* serpin controls Toll-mediated morphogenesis in the embryo and the phenol-oxidase cascade in adults [54],[55]. *Spn28D* (*CG7219*) also regulates the phenol-oxidase cascade [56], while *Spn77Ba* regulates tracheal melanization, which also can trigger systemic expression of Drosomycin via the Toll pathway [57]. In addition, the *Spn42Da* transcript inhibits furin, which is involved in the maturation of secreted proteins [58].

In mammals, serpins are removed from circulation by endocytosis and degraded in the liver as inactive serpin/proteinase complexes. This aspect of serpin metabolism, however, has not been studied previously in *Drosophila*. In this study, we identify the mechanism of serpin clearance by endocytosis in the garland and pericardial cells. These cells are known to take up ferritin [59] and GFP [60] and have been suggested to be homologous to mammalian reticulo-endothelial cells [41],[43] or nephrocytes [34],[40],[61]. As in mammals, serpin turnover in *Drosophila* is extremely rapid, so that we were unable to detect the immune-response serpins Nec and Spn27A under normal conditions. However, freezing the pinching-off of endocytotic vesicles, using the *shi^ts1^* mutation, allows us to detect serpin uptake. Endocytosed Nec is sorted, first to Rab5-positive early-endosomes and then to Rab7-positive late-endosomes. Disrupting these steps by expression of dominant negative *UAS*-*Rab^5S43^* or *UAS*-*Rab7^Q67L^*, leads to accumulation of Nec-positive vesicles. Similarly, co-localisation of anti-Nec and anti-HRS antibody staining indicates that the serpin is present in early endosomes in the ubiquitin-dependent sorting pathway; while anti-Nec and anti-Fab1 confirms that the serpin is destined for lysosomal degradation. Blocking late-endosome/lysosome fusion using HOPS-complex mutants causes accumulation of Nec-positive endosomes/MVB, indicating that Nec sorting to MVB is required for lysosomal delivery. Co-localization of anti-Nec and anti-Lamp1 antibody staining confirms that Nec is delivered to lysosomes for degradation, while the absence of Nec staining in Rab11-positive vesicles indicates that none of the serpin is recycled from MVB to the haemolymph. In summary, Nec is cleared from the haemolymph and sorted through MVB, via the ubiquitin-dependent pathway, to lysosomes for degradation.

*In vitro* studies in mammals have shown that different members of LDLR family have different binding specificities to different native serpins and serpin/proteinase complexes [15],[62]. In this study, we have shown that LpR1 is the Nec trafficking receptor *in vivo*, but that neither LpR1 nor LpR2 traffics Spn27A. By analogy to mammalian systems, Nec is probably taken up by LpR1 as a complex with its target proteinase. In addition, pre-digestion of Nec with PPE increases Nec uptake in garland cells that are not deficient for the LpR1 receptor. Our results establish that active trafficking of Nec from the haemolymph can modulate the immune response. Nec clearance is extremely rapid, but deletion of the *LpR1* gene sensitises the immune response: *nec* transcript levels decrease and *Drs* transcript levels increase. These results imply a regulatory feedback loop at the transcriptional level. In this context, it is significant that LpR1 appears to bind the non-inhibitory serpin/proteinase complex, in preference to the native Nec serpin. Clearance of the serpin/protease complex through the athrocytes appears to compete with a regulatory feedback loop affecting *nec* transcription.

In summary, we establish that the Nec serpin is taken-up via LpR1 from the haemolymph and degraded in the garland and pericardial athrocytes.

## Materials and Methods

### Fly strains and crosses

The following *Drosophila* stocks were obtained from the Bloomington Stock Centre and described in FlyBase: s*hi^ts1^*, *PBac(PB)LpR1^c04916^*, *hk^1^*, *dor^8^*, *dor^1^*, *car^1^*, *Df(2R)STI* and *nec^2^*. The *nec^19^* stock was described in [63] and *Df(2R)pk-sple-51* in [64]. The chromosomal deletion for *nec* was a trans heterozygous combination of *Df(2R)pk-sple-51/Df(2R)STI*, which completely lacks the *nec* and *pk* transcripts. *UAS-Rab^5S43^* and *UAS-Rab7^Q67L^* are described in [65]. We used two homozygous viable deletions that remove the individual *LpR1* transcripts, *Df(3R)lpr1* and *Df(3R)lpr2*, and a third homozygous viable deletion, *Df(3R)lpr1/2*, which removes both transcripts together, without including other genes (J. Culi unpublished data). The *dot-Gal4 UAS-GFP* stock is described in [36] and the fat-body *Gal4* driver, *c564-Gal4* was from Bloomington (*GawB-c564*). Stocks were grown on standard cornmeal/agar medium at 25°C, except those containing the *shi^ts1^* mutation, which were grown at 18°C.

### *M. luteus* infection

The Gram-positive bacterium *Micrococcus luteus*, strain CIP A270, was used to activate the Toll-mediated immune response. Adult flies were challenged by septic injury with a thin tungsten needle dipped into a concentrated *M. luteus* culture. Flies were collected after 6 h (for Nec assays) or 24 h (for Drs response). Larvae were infected by incubation on a Petri dish carrying an overnight culture of *M. luteus*.

### Immunostaining

Third instar larvae and adults were dissected in PBS at 20°C and fixed in 4% PFA solution at 37°C or at 4°C. Immunohistochemistry was performed according to [66]. Non-specific background staining was reduced by adding BSA (1%) to the wash solutions. The following polyclonal guinea pig antibodies were used in this study: HRS at 1∶100 dilution [26], anti-Fab1 at 1∶200 dilution and anti-Lamp1 at 1∶500 dilution [27], LpR1 at 1∶100 dilution and LpR2 at 1∶100 dilution (J. Culi unpublished data). Polyclonal rabbit antibodies used were: anti-Nec at 1∶500 dilution [63], anti-Spn27 at 1∶500 dilution [55]; anti-Rab7 at 1∶2000 dilution [67], anti-Rab5 at 1∶50 dilution [68]. Monoclonal mouse antibodies were purchased from BD Transduction laboratories∶anti-AP50 at 1∶25 dilution (to mark clathrin-coated vesicles) [69] and anti-Rab11 at 1∶100 dilution [70]. Anti-HRP-Cy2 at 1∶100 dilution, to detect garland cell membranes (Jackson ImmunoResearch Laboratories). Alexa Fluor 488, Alexa Fluor 568 and Alexa Fluor 633, conjugated secondary antibodies at 1∶200 dilution (Jackson ImmunoResearch Laboratories). Phalloidin at 1∶800 dilution was used to detect Actin (Sigma-Aldrich) and DAPI at 1∶5000 dilution (Sigma-Aldrich) to stain DNA. For double staining the first antibody (followed by its specific secondary antibody) was eluted, blocked and rinsed before addition of the second antibody.

### Confocal microscopy

A Leica confocal microscope and software were used. Images were processed using Adobe Photoshop.

### Complex formation

The Nec core serpin, ΔN-Nec was incubated with PPE in PBS as previously described [52]. Porcine pancreatic elastase (PPE) was purchased from Sigma. ΔN-Nec was purified with the IMPACT-NT protein purification system [52].

### Endocytotic assay

Garland cells were dissected and transferred to PBS solution on ice. Garland cell serpin-uptake studies were performed by incubating cells in PBS solution containing 0.2 mg/ml purified ΔN-Nec solution or 0.2 mg/ml ⊗N-Nec-PPE complex. After 20 minutes incubation at room temperature, cells were fixed and stained as described above.

### Whole-mount *in-situ* hybridisation

The Nec antisense DIG-labelled RNA probe (cDNA nucleotides 586–1450) was prepared according to the manufacturer's instructions (Roche Biochemical).

### dsRNA preparation

Templates for dsRNA preparation were obtained by PCR amplification between two T7 promoter sequences. Fragments for each gene were as follows: *CG8909* (nucleotides 842–1537), *CG33087* (12870–13136), *CG12139* (1758–2346), *LpR2* (3133–3748), *LpR1* (2521–3033) and *arrow* (1572–2144). Single-stranded RNAs were synthesized and precipitated with the MEGAscript T7 transcription kit (Ambion). dsRNA was dissolved in injection buffer (0,1 mM sodium phosphate, pH 6.8; 5 mM KCL).

### cDNA preparation and quantitative Real Time–PCR

Total RNA was extracted using RNAeasy Mini Kit (Quiagen) according to the manufacturer's instructions. For qRT–PCR, 1 µg of RNA was reverse-transcribed using ThermoScript RT-PCR (Invitrogen). cDNA was quantified by real-time PCR using iQ SYBERGreen Supermix (Biorad), with the iCycler (Bio-Rad) thermocycler. Probes were normalized against a ribosomal protein 49 (rp49) control. Samples were run three times and the relative levels of a given mRNA were normalized by cycling threshold analysis (ΔCT).

## Supporting Information

Figure S1 qRT-PCR shows low levels of *nec* expression during embryonic and moderate levels during larval development. Expression levels are somewhat higher in unchallenged adults than third instar larvae, but reach very similar levels in both stages 6 h post infection. Samples: embryonic (mixed stages, 0–24 h), larval instars L1, L2, and L3, pupae P1 (12–48 h), P2 (48–96 h), Adult (1 day post hatch).
(0.07 MB TIF)

Figure S2 *nec* tissue *in situs* in infected larvae. (A) *In situ* hybridisation shows cytoplasmic *nec* transcript (arrow) in fat-body cells of wild-type larvae. (B) *Df(2R)nec^−^* (transcript null) fat-body cells show weak background staining, but lack strong cytoplasmic staining. (C) No *nec* transcript is detected in wild-type larval garland cells (white arrows). Bar is 100 micrometers.
(1.44 MB TIF)

Figure S3 Nec antibody staining in the garland cells with *nec^dsRNAi^* knockdown, 6 h post-infection. (A) Merge shows that Nec staining remains strong despite *dot-Gal4* driven knockdown, monitored by *UAS-GFP* (in *sh^its^*; *UAS-Nec^dsRNAi^*; *dot-Gal4 UAS-GFP* larvae). (B) Nec channel. (C) GFP channel. (D) DAPI channel. Nec (red), GFP (green) and DAPI (blue).
(1.54 MB TIF)

Figure S4 RNAi knockdown of *nec* in the fat-body eliminates detectable Nec protein uptake in garland cells (white arrow). Nec (red), Actin (green) and DAPI (blue).
(1.03 MB TIF)

Figure S5 Time course of Nec uptake in garland cells following infection in *shi^ts1^* larvae. Strongest Nec staining was detected 6–8 h post infection. Nec (red), Actin (green) and DAPI (blue). All pictures are captured with the same settings and the same laser intensity.
(3.87 MB TIF)

Figure S6 Effect of silencing *Drosophila* LDLR-family homologues on drosomycin transcript levels, 24 hr post infection. Silencing of *CG8909*, *LRP1-like*, *Megalin-like* and *arrow* do not affect *Drs* transcript levels significantly compared to control wild-type flies. *LpR1* silencing increases Drs transcript (200%), while *LpR2* silencing causes a decrease in Drs transcript (68%).
(0.10 MB TIF)

Figure S7 Effect of homozygous deletions of *LpR* genes on *Drs* and *nec* transcript levels, either 24 hr post infection or without infection. A) Levels of *nec* transcript in infected *Df(3R)lpr1*, *Df(3R)lpr2* and *Df(3R)lpr1/2* adults, compared to wild-type control flies. B) Levels of *Drs* transcript in infected *Df(3R)lpr1*, *Df(3R)lpr2* and *Df(3R)lpr1/2* adults, compared to wild-type control flies. C) Levels of *Drs* transcript in uninfected *Df(3R)lpr1* adults, compared to infected and uninfected wild-type control flies. Deletion of the LpR1 transcript increases Drs transcript levels 1030% in compared to wild-type, in uninfected adults.
(0.82 MB TIF)

Figure S8 LpR2 and Nec co-localize in garland and pericardial cells. (A–D) larval, (A′–D′) adult garland cells, (A″–D″) larval and (A‴–D‴) adult pericardial cells, 6 h post infection. (A, A′, A″ and A‴) Merge shows LpR2 and Nec co-localizing (yellow) in endosomes. (B, B′, B″ and B‴) Nec channel. (C, C′, C″ and C‴) LpR2 channel. (D, D′, D″ and D‴) DAPI channel. Nec (red), LpR2 (green), and DAPI (blue).
(4.61 MB TIF)
