# Supplementary figures and images for: Uptake of the Necrotic Serpin in Drosophila melanogaster via the Lipophorin Receptor-1
Source: PLoS Genet. 2009 Jun 26;5(6):e1000532. doi: 10.1371/journal.pgen.1000532 (PMC2694266; doi:10.1371/journal.pgen.1000532)

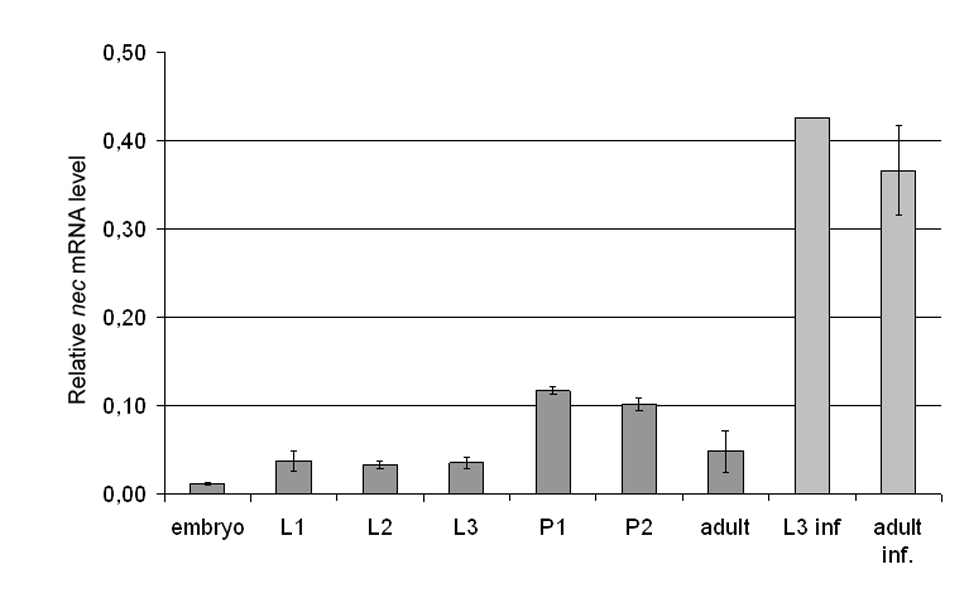

Supplement: Figure S1 — qRT-PCR shows low levels of nec expression during embryonic and moderate levels during larval development. Expression levels are somewhat higher in unchallenged adults than third instar larvae, but reach very similar levels in both stages 6 h post infection. Samples: embryonic (mixed stages, 0–24 h), larval instars L1, L2, and L3, pupae P1 (12–48 h), P2 (48–96 h), Adult (1 day post hatch). (0.07 MB TIF) [file pgen.1000532.s001.tif]

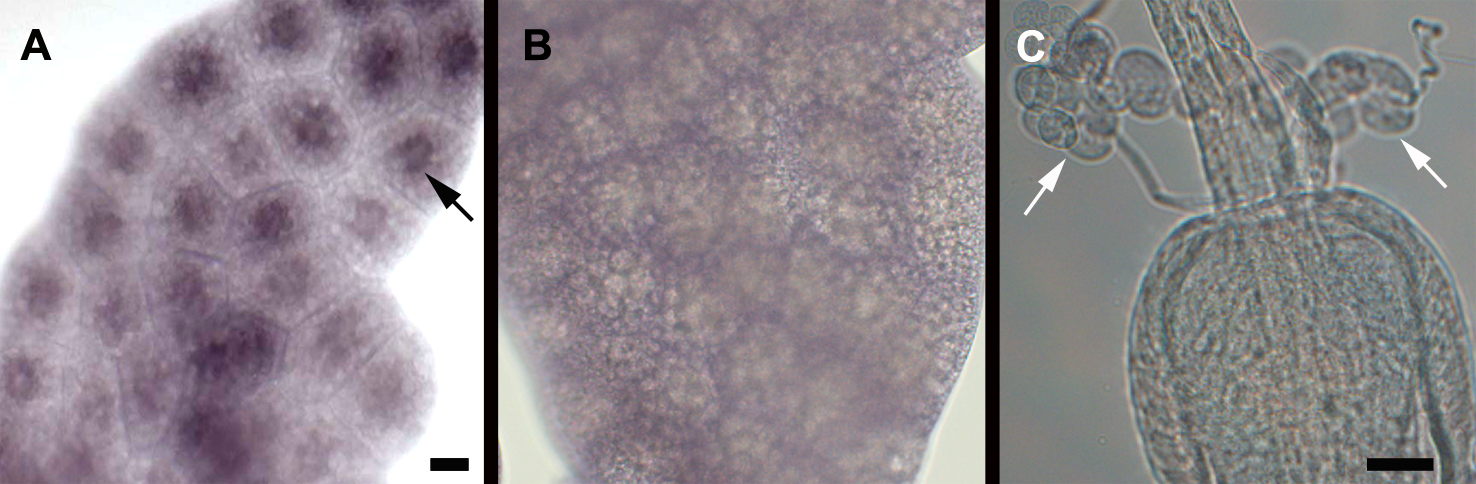

Supplement: Figure S2 — nec tissue in situs in infected larvae. (A) In situ hybridisation shows cytoplasmic nec transcript (arrow) in fat-body cells of wild-type larvae. (B) Df(2R)nec− (transcript null) fat-body cells show weak background staining, but lack strong cytoplasmic staining. (C) No nec transcript is detected in wild-type larval garland cells (white arrows). Bar is 100 micrometers. (1.44 MB TIF) [file pgen.1000532.s002.tif]

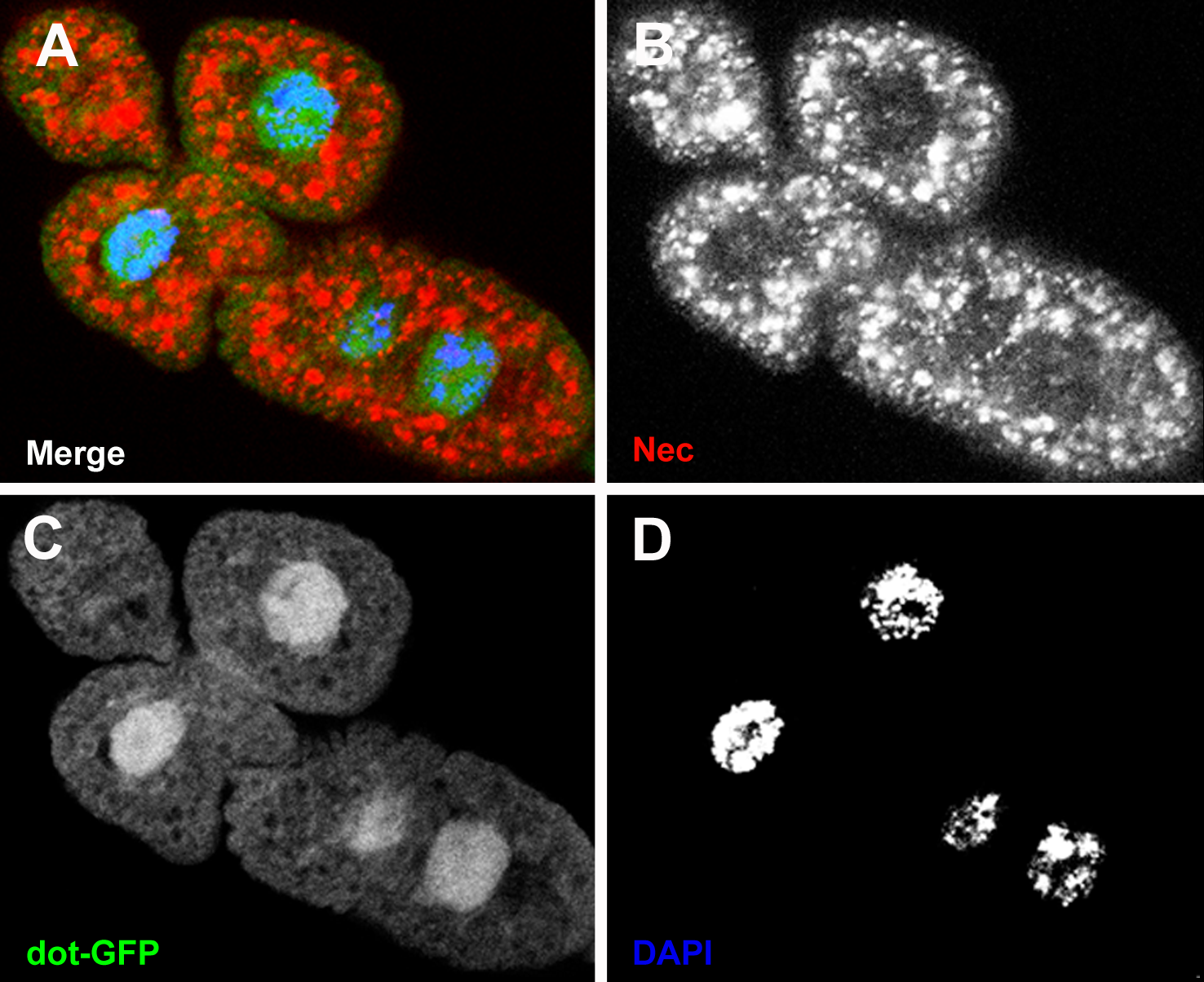

Supplement: Figure S3 — Nec antibody staining in the garland cells with necdsRNAi knockdown, 6 h post-infection. (A) Merge shows that Nec staining remains strong despite dot-Gal4 driven knockdown, monitored by UAS-GFP (in shits; UAS-NecdsRNAi; dot-Gal4 UAS-GFP larvae). (B) Nec channel. (C) GFP channel. (D) DAPI channel. Nec (red), GFP (green) and DAPI (blue). (1.54 MB TIF) [file pgen.1000532.s003.tif]

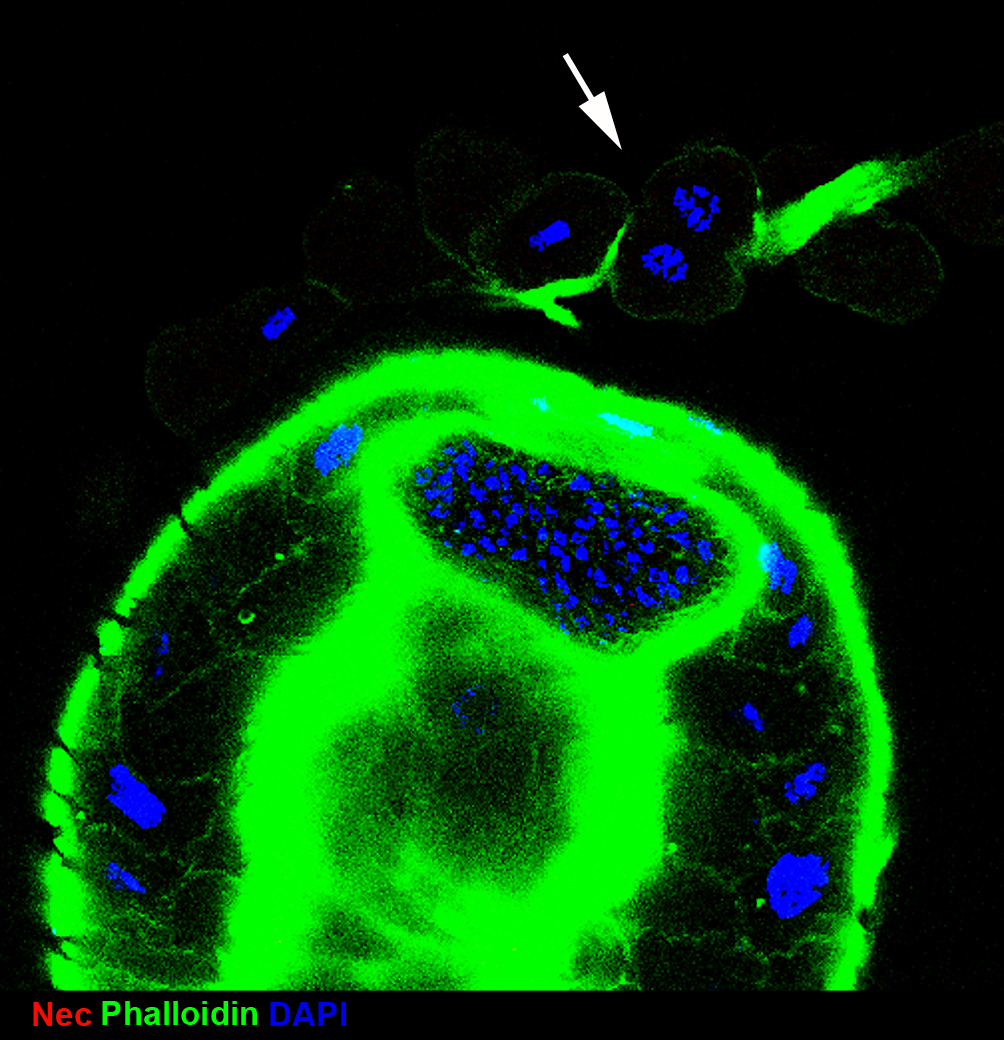

Supplement: Figure S4 — RNAi knockdown of nec in the fat-body eliminates detectable Nec protein uptake in garland cells (white arrow). Nec (red), Actin (green) and DAPI (blue). (1.03 MB TIF) [file pgen.1000532.s004.tif]

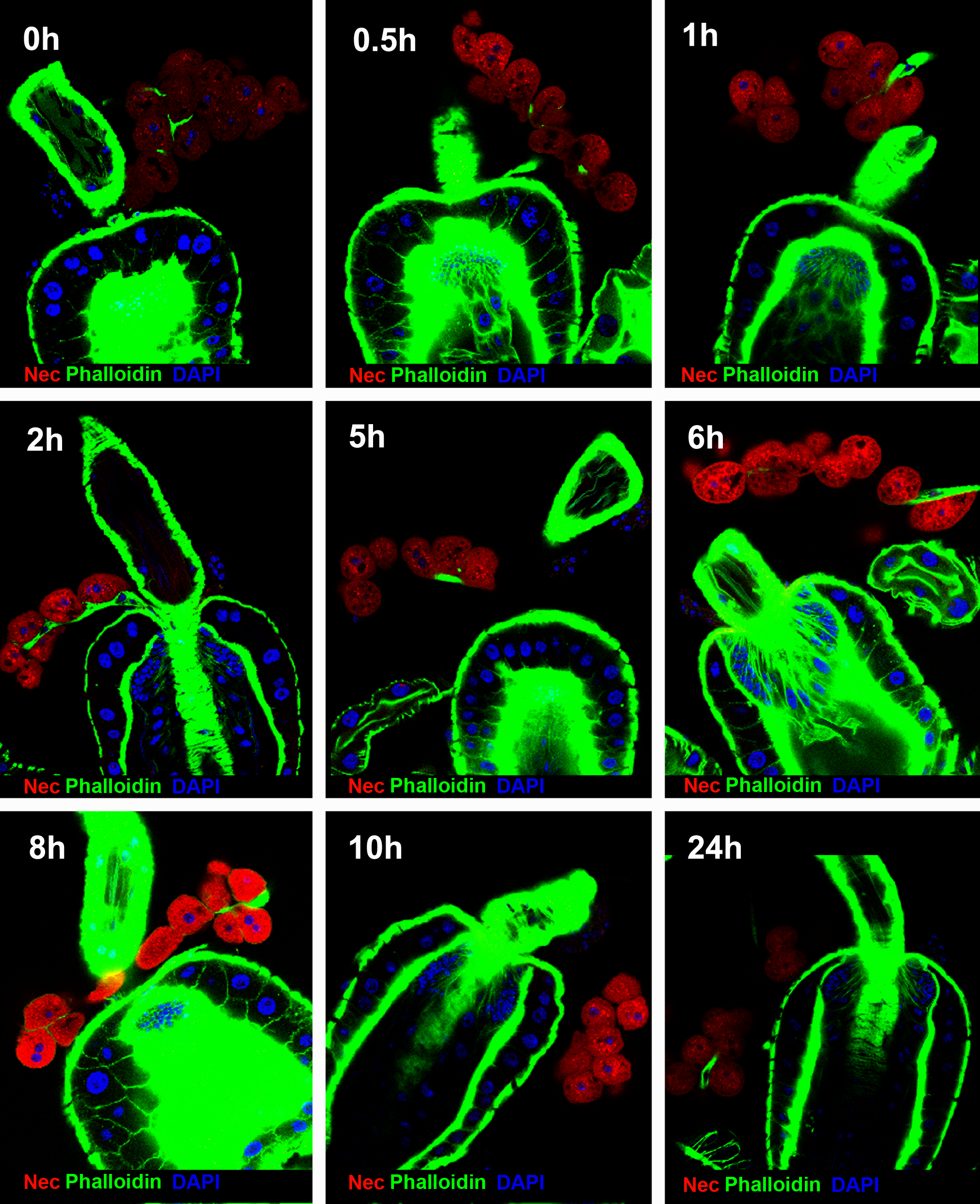

Supplement: Figure S5 — Time course of Nec uptake in garland cells following infection in shits1 larvae. Strongest Nec staining was detected 6–8 h post infection. Nec (red), Actin (green) and DAPI (blue). All pictures are captured with the same settings and the same laser intensity. (3.87 MB TIF) [file pgen.1000532.s005.tif]

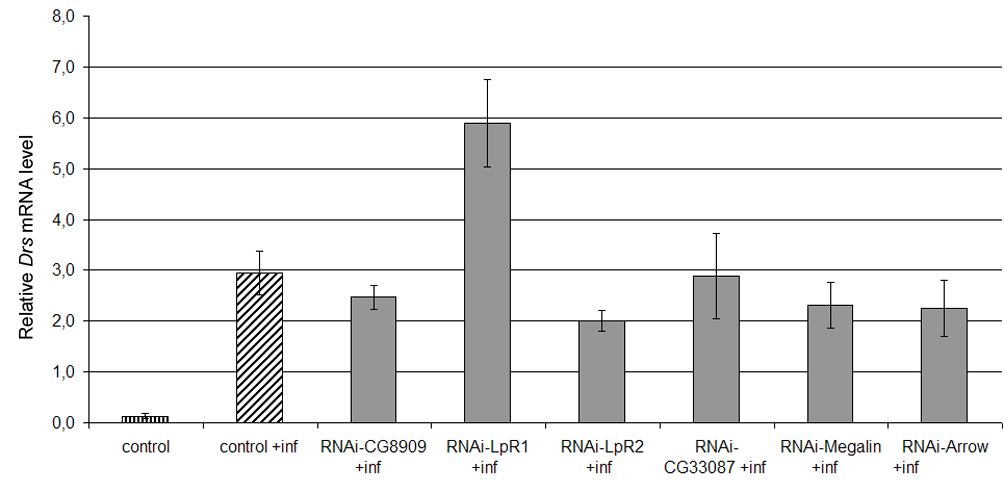

Supplement: Figure S6 — Effect of silencing Drosophila LDLR-family homologues on drosomycin transcript levels, 24 hr post infection. Silencing of CG8909, LRP1-like, Megalin-like and arrow do not affect Drs transcript levels significantly compared to control wild-type flies. LpR1 silencing increases Drs transcript (200%), while LpR2 silencing causes a decrease in Drs transcript (68%). (0.10 MB TIF) [file pgen.1000532.s006.tif]

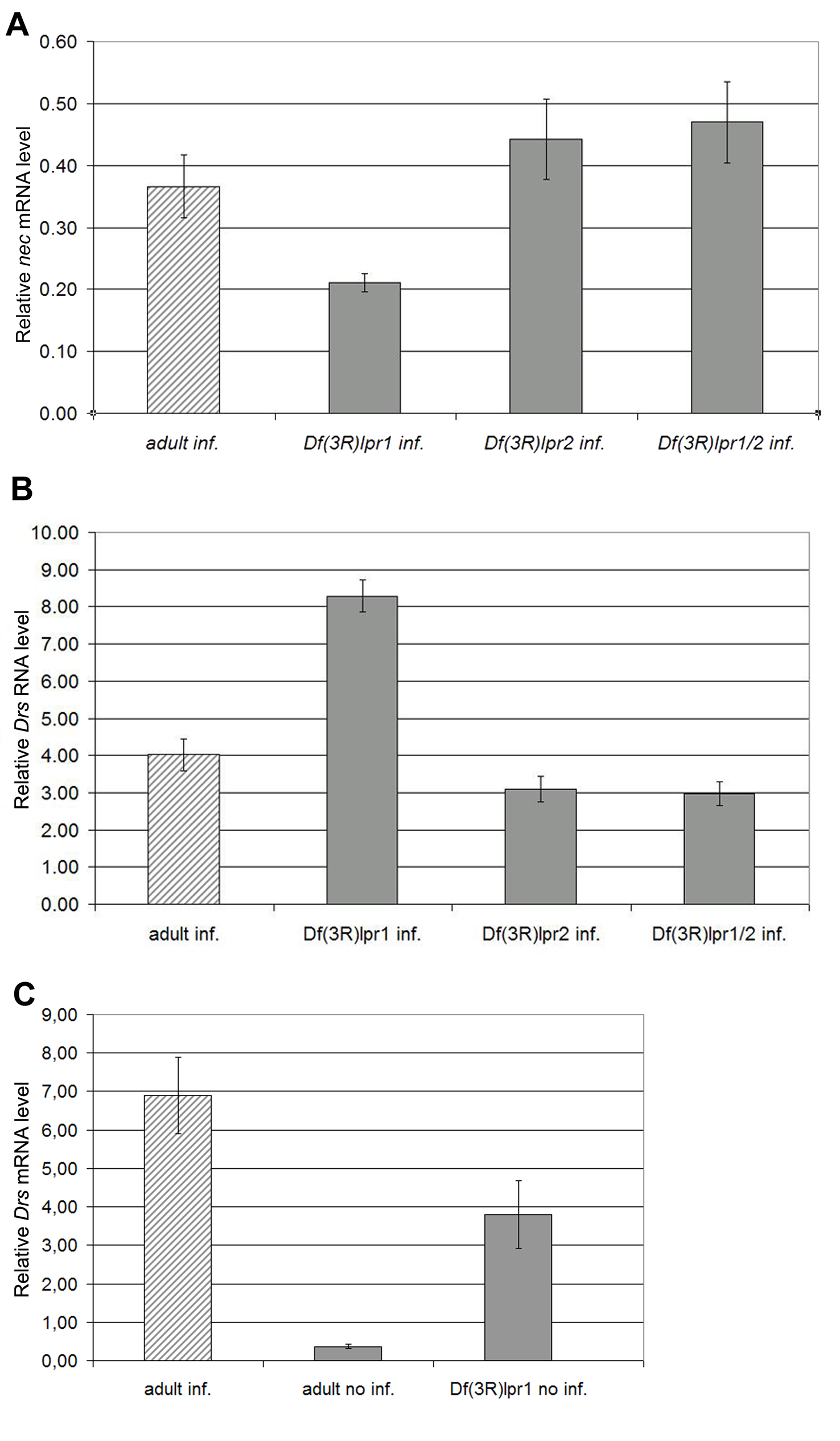

Supplement: Figure S7 — Effect of homozygous deletions of LpR genes on Drs and nec transcript levels, either 24 hr post infection or without infection. A) Levels of nec transcript in infected Df(3R)lpr1, Df(3R)lpr2 and Df(3R)lpr1/2 adults, compared to wild-type control flies. B) Levels of Drs transcript in infected Df(3R)lpr1, Df(3R)lpr2 and Df(3R)lpr1/2 adults, compared to wild-type control flies. C) Levels of Drs transcript in uninfected Df(3R)lpr1 adults, compared to infected and uninfected wild-type control flies. Deletion of the LpR1 transcript increases Drs transcript levels 1030% in compared to wild-type, in uninfected adults. (0.82 MB TIF) [file pgen.1000532.s007.tif]

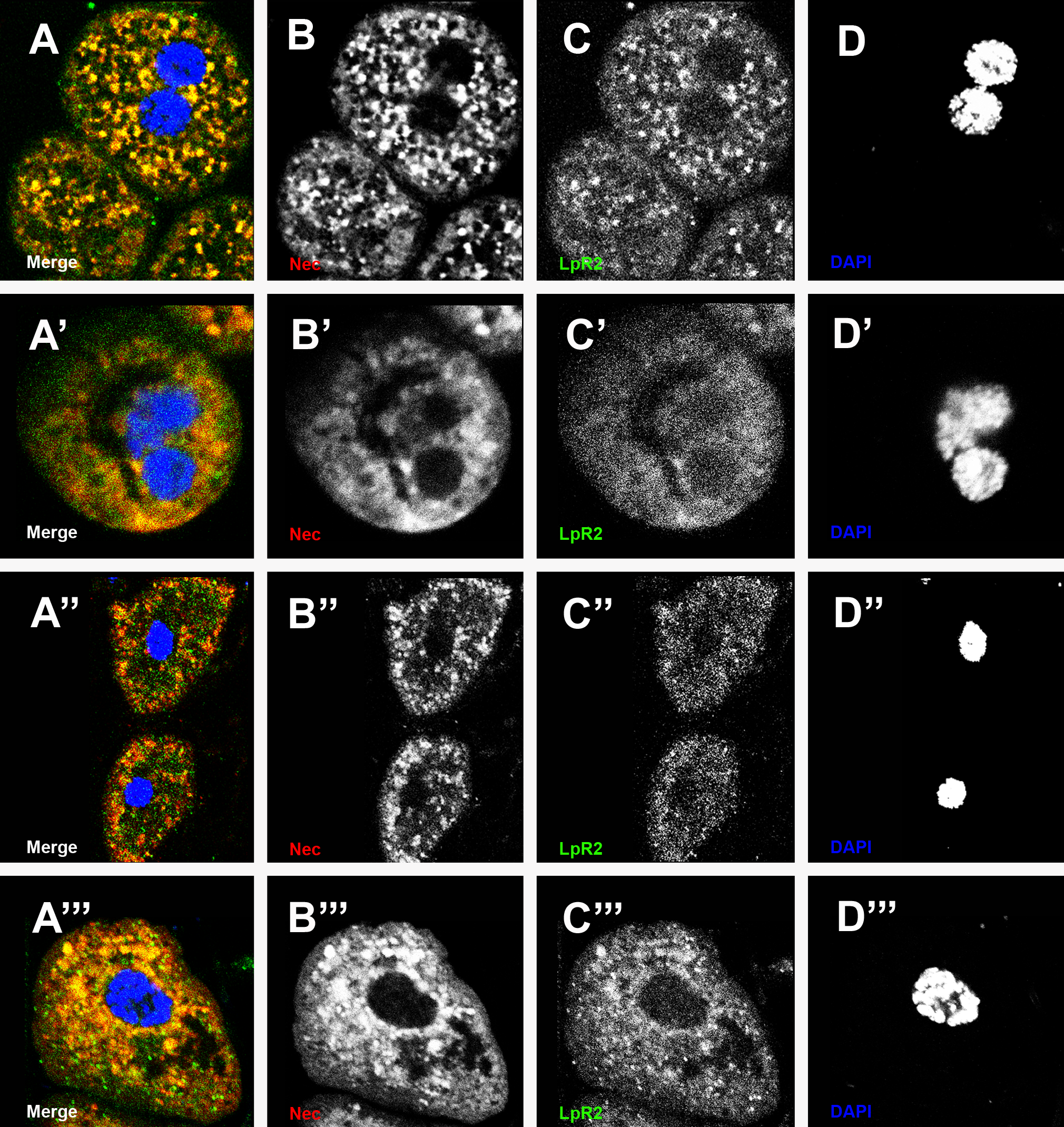

Supplement: Figure S8 — LpR2 and Nec co-localize in garland and pericardial cells. (A–D) larval, (A′–D′) adult garland cells, (A″–D″) larval and (A‴–D‴) adult pericardial cells, 6 h post infection. (A, A′, A″ and A‴) Merge shows LpR2 and Nec co-localizing (yellow) in endosomes. (B, B′, B″ and B‴) Nec channel. (C, C′, C″ and C‴) LpR2 channel. (D, D′, D″ and D‴) DAPI channel. Nec (red), LpR2 (green), and DAPI (blue). (4.61 MB TIF) [file pgen.1000532.s008.tif]
